# Integrated Transcriptome–Metabolome Analysis Reveals the Flavonoids Metabolism Mechanism of Maize Radicle in Response to Low Temperature

**DOI:** 10.3390/plants14192988

**Published:** 2025-09-26

**Authors:** Yi Dou, Wenqi Luo, Yifei Zhang, Wangshu Li, Chunyu Zhang, Yanjie Lv, Xinran Liu, Song Yu

**Affiliations:** 1 College of Agriculture, Heilongjiang Bayi Agricultural University, Daqing 163319, China; bynddy@163.com (Y.D.); byndlwq@163.com (W.L.); liwangshu07@163.com (W.L.); 13684592065@163.com (C.Z.); lxr12132002@163.com (X.L.); byndys@163.com (S.Y.); 2 Key Laboratory of Low-Carbon Green Agriculture in Northeastern China, Ministry of Agriculture and Rural Affairs, Daqing 163319, China; 3 Institute of Agricultural Resources and Environment, Jilin Academy of Agricultural Sciences, Changchun 130033, China; lvyanjie_1977@163.com

**Keywords:** low-temperature stress, maize radicle, transcriptome, metabolome, integration analysis, flavonoid metabolic regulation pathway

## Abstract

The Northeast region in China is a major maize-producing area; however, low-temperature stress (TS) limits maize (*Zea mays* L.) seed germination, affecting population establishment and yield. In order to systematically explore the regulation mechanism of maize radicle which is highly sensitive to low-temperature environment response to TS, seeds of ZD958 and DMY1 were used to investigate germination responses under 15 °C (control) and 5 °C (TS) conditions. Phenotypic, physiological, transcriptomic, and metabolomic analyses were conducted on the radicles after 48 h of TS treatment. TS caused reactive oxygen species (ROS) imbalance and oxidative damage in radicle cells, inhibiting growth and triggering antioxidant defenses. Integrated transcriptomic and metabolomic analyses revealed that flavonoid metabolism may play a pivotal role in radicle responses to TS. Compared with the control treatment, ZD958 and DMY1 under TS treatment significantly increased (*p* < 0.01) the total flavonoid content, total antioxidant capacity, 4-coumarate-CoA ligase activity, and dihydroflavonol 4-reductase activity by 15.99% and 16.01%, 18.41% and 18.54%, 63.54% and 31.16%, and 5.09% and 7.68%, respectively. Despite genotypic differences, both followed a shared regulatory logic of “low-temperature signal-driven—antioxidant redirection—functional synergy.” This enabled ROS scavenging, redox balance, and antioxidant barrier formation, ensuring basal metabolism and radicle development.

## 1. Introduction

Maize (*Zea mays* L.) is a globally important multi-purpose crop used for food, feed, and industrial raw materials [1]. It has the largest planting area in China, accounting for more than one-third of China’s total grain production. The Northeast region of China is also a major maize-producing area, and the region’s sustained high-yield, efficient maize cultivation plays a vital role in stabilizing regional food supply levels and agricultural economic development [2]. Reasonable planting density can optimize the spatial layout of the population and coordinate resource competition between individuals and the population, thereby improving the photosynthetic efficiency and resource utilization efficiency, and ultimately increasing maize yield [3]. Seed germination is the initial stage of crop growth and development, which in turn affects yield and quality. Its success directly affects the quality of seedling establishment and relates to the subsequent productivity and production efficiency of the crop population [4,5]. However, owing to global climate change, unfavorable meteorological conditions such as seasonal low temperatures and cold damage during the spring season have become more frequent in northeastern China, particularly in high-latitude cold regions [6]. These conditions often result in delayed maize germination, reduced emergence and survival rates, and notably poorer uniformity [7]. These factors make it difficult to form high-quality dense plant populations, and has now become one of the key issues affecting the efficient mechanized production of maize throughout its growth cycle in the high-latitude cold regions of northeastern China, severely constraining the sustained increase in maize yields and the high-quality development of the agricultural economy.

As one of the primary abiotic stresses, low-temperature cold damage can cause excessive accumulation of reactive oxygen species (ROS) in plant cells, leading to membrane lipid peroxidation reactions and DNA, RNA, and protein damage [8], as well as inhibiting the function of the mitochondrial electron transport chain, reducing ATP synthesis efficiency, and inhibiting plant growth and development [9]. To mitigate the adverse effects of low-temperature environments, plant cells have developed various repair mechanisms, among which antioxidant system activation is a core defense strategy [10]. As a key component of the antioxidant defense system, flavonoids, including isoflavones, flavonols, anthocyanidins, flavanols, flavanones, chalcones, and dihydrochalcones, are essential natural secondary metabolites widely distributed in plants [11]. Their unique C6-C3-C6 benzene ring structure enables them to act as reducing agents in certain reactions. Their antioxidant effects are exerted through a multi-dimensional synergistic mechanism, including reducing free radical formation, scavenging stress-induced ROS, inhibiting the activity of superoxide anion (O_2_^−^)-producing oxidases, and activating antioxidant enzymes [12], thus protecting plants from UV radiation, microbial damage, and oxidative stress-induced injury [13,14]. Temperature is the primary environmental factor regulating secondary metabolites in plants, and stress induced by temperatures between 4 and 10 °C leads to flavonoid and terpenoid accumulation [15]. Transcriptomic and metabolomic studies have indicated that under low-temperature stress (TS), the application of exogenous melatonin can alleviate damage to *Elymus nutans* Griseb. by enhancing the activity of antioxidant enzymes and increasing glycyrrhizin and delphinidin accumulation [16]. Furthermore, the differential synthesis and accumulation of 13 flavonoids (phloretin, epicatechin and quercetin, etc.) in the needles of wild-type *Cryptomeria fortunei* is one of the important mechanisms for its superior cold resistance [17]. Compared to cold-sensitive varieties, cold-tolerant alfalfa varieties actively upregulate the expression of genes related to flavonoid biosynthesis enzymes, such as chalcone synthase, anthocyanin dioxygenase, and flavonoid 3′-monooxygenase in leaves, synthesizing more flavonoids to counteract the adverse effects of low temperatures [18].

As one of the primary components of the seed embryo, the radicle is highly sensitive to low-temperature environments from seed germination to seedling development. In numerous studies on rice [19], maize [20], and halophytes [21], the radicle has been used as an important indicator in low-temperature tolerance variety screening and evaluation, molecular-assisted breeding, and innovation in stress-tolerant cultivation techniques. Although previous studies have investigated the mechanisms by which maize radicles respond to abiotic stress [22,23,24,25,26,27], the mechanisms underlying radicle responses to TS during maize germination remain unknown. Therefore, in order to systematically explore the regulation mechanism of maize radicle response to TS, seeds of two varieties Zhengdan958 (ZD958) and Demeiya1 (DMY1), primarily cultivated in the high-latitude cold regions of Northeastern China were used to investigate germination responses under 15 °C (control, CT) and 5 °C (TS) conditions. Phenotypic, physiological, transcriptomic, and metabolomic analyses were conducted on the radicles after 48 h of TS treatment. This study deepens the understanding of the physiological basis of maize radicles adapting to TS by revealing the metabolic regulatory pathway of flavonoids. It lays a theoretical foundation for improving the low-temperature tolerance of maize seeds during germination and optimizing the corresponding cultivation regulation techniques in cold regions.

## 2. Results

### 2.1. Effect of TS on the Phenotypic Characteristics of Maize Radicles

**Figure 1:**
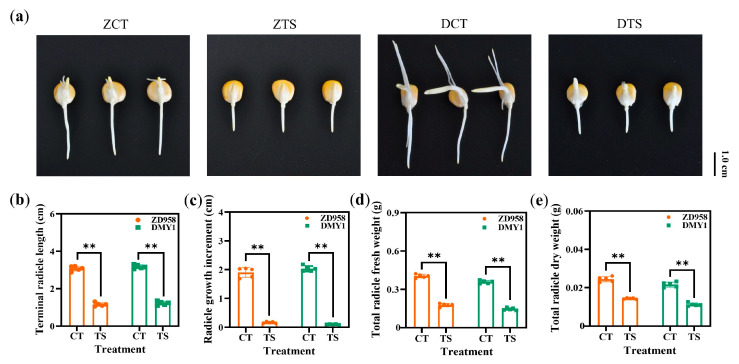
Differences in maize radicle morphology and growth performance under different treatments. (**a**) Morphological characteristics of ZD958 and DMY1 seeds under CT or TS conditions; (**b**) terminal length of maize radicles under CT or TS treatment conditions for different genotypes; (**c**) growth increment of maize radicles under CT or TS treatment conditions for different genotypes; (**d**) total fresh weight of maize radicles under CT or TS treatment conditions for different genotypes; (**e**) total dry weight of maize radicles under CT or TS treatment conditions for different genotypes. CT: control treatment; TS: low-temperature stress. Data represent the mean ± standard error (SE) of five replicates (*n* = 5). **: *p* < 0.01.

Under TS conditions, significant (*p* < 0.01) changes in the growth of ZD958 and DMY1 radicles were observed (Figure 1a). After 48 h of treatment, the terminal radicle length of ZD958 under CT treatment (ZCT) and DMY1 under CT treatment (DCT) increased by 1.90 cm and 2.03 cm, respectively, whereas those of ZD958 under TS treatment (ZTS) and DMY1 under TS treatment (DTS) increased by only 0.17 cm and 0.10 cm, respectively. This resulted in a reduction of 57.14% to 64.62% in the terminal radicle length of the TS treatment compared to the CT treatment (Figure 1b,c). Additionally, compared to the CT treatment, the fresh weight (FW) and dry weight (DW) of the embryonic roots in the ZTS and DTS decreased by 55.34% and 41.44%, and 58.88% and 48.15%, respectively, with all differences reaching extremely significant levels (*p* < 0.01; Figure 1d,e).

### 2.2. Effect of TS on Physiological and Biochemical Indicators of Maize Radicles

**Figure 2:**
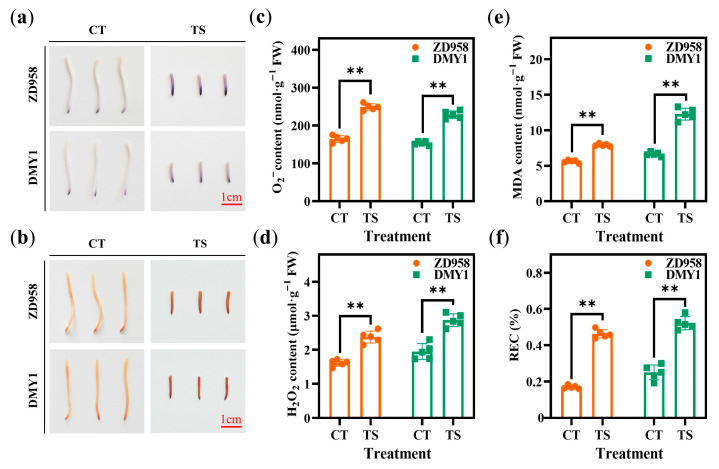
Histochemical staining and physiological index changes in maize radicles under different treatments. (**a**) Staining of maize radicles of different genotypes under CT or TS treatment conditions using NBT; (**b**) staining of maize radicles of different genotypes under CT or TS treatment conditions using DAB; (**c**) O_2_^−^ content of maize radicles of different genotypes under CT or TS treatment conditions; (**d**) H_2_O_2_ content in maize radicles of different genotypes under CT or TS treatment conditions; (**e**) MDA content in maize radicles of different genotypes under CT or TS treatment conditions; (**f**) REC of maize radicles of different genotypes under CT or TS treatment conditions. Data represent the mean ± SE of five replicates (*n* = 5). **: *p* < 0.01.

**Figure 3:**
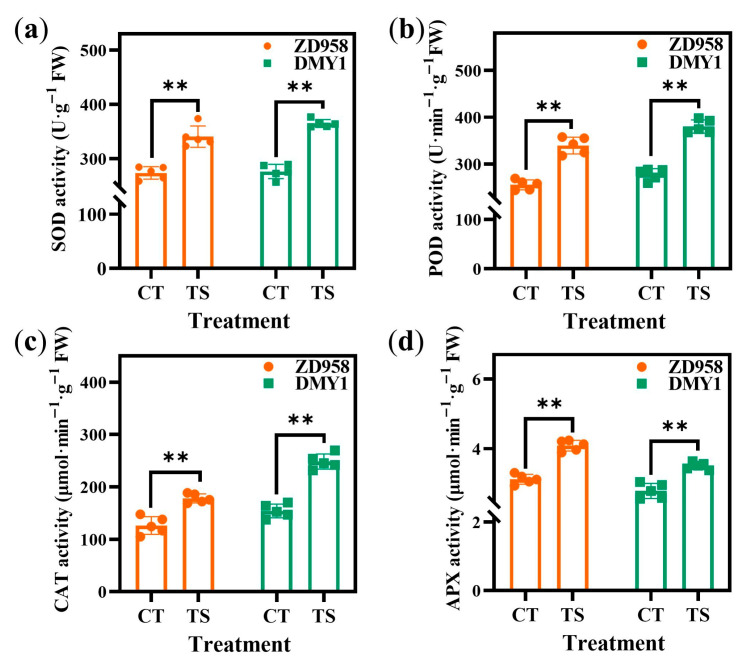
Changes in antioxidant enzyme activity in maize radicles under different treatments. (**a**) SOD activity; (**b**) POD activity; (**c**) CAT activity; and (**d**) APX activity. SOD: superoxide dismutase; POD: peroxidase; CAT: catalase; APX: ascorbate peroxidase. Data represent the mean ± SE of five replicates (*n* = 5). **: *p* < 0.01.

After staining the radicles of different varieties using nitrotetrazolium blue chloride (NBT) and 3,3′-diaminobenzidine (DAB), the color of the TS treatment was significantly darker than that of the CT treatment (Figure 2a,b). Simultaneously, compared to O_2_^−^, H_2_O_2_, and malondialdehyde (MDA) levels in CT-treated radicles, ZTS increased these levels by 51.43%, 47.14%, and 43.38%, respectively, whereas DTS increased them by 49.10%, 47.40%, and 84.90%, respectively (Figure 2c–e). Similarly, the relative electrical conductivity (REC) of ZTS and DTS radicles increased by 173.24% and 108.30%, respectively, compared to CT treatment across all varieties (Figure 2f). Additionally, superoxide dismutase (SOD), peroxidase (POD), catalase (CAT), and ascorbate peroxidase (APX) activities increased under TS treatment, with increases of 27.85–34.72%, 29.91–32.46%, 56.00–60.84%, and 27.71–27.94, respectively, compared to CT treatment (Figure 3). The differences in these physiological indicators were highly significant (all *p* < 0.01).

### 2.3. RNA-Seq and Differentially Expressed Gene Analysis of Maize Radicles Under TS

**Figure 4:**
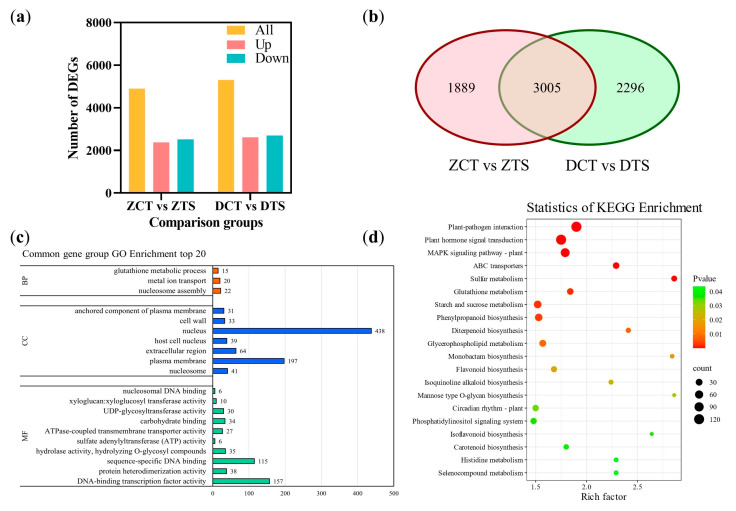
DEGs in different treatment comparison groups and their GO and KEGG enrichment analysis. (**a**) Number of DEGs in the comparison groups “ZCT vs. ZTS” and “DCT vs. DTS”; (**b**) Number of unique and common DEGs between “ZCT vs. ZTS” and “DCT vs. DTS”; (**c**) Top 20 GO terms analysis of common DEGs between “ZCT vs. ZTS” and “DCT vs. DTS”; (**d**) Top 20 KEGG pathways of the common DEGs between “ZCT vs. ZTS” and “DCT vs. DTS”; In the bubble plot, the x-axis represents the enrichment factor, and the y-axis represents the KEGG pathway, with colors ranging from red to green indicating *p*-values from small to large. ZCT: control treatment of ZD958; ZTS: TS treatment of ZD958; DCT: control treatment of DMY1; DTS: TS treatment of DMY1; MF: molecular function; CC: cellular component; GO, Gene Ontology; KEGG: Kyoto Encyclopedia of Genes and Genomes.

This study obtained a total of 90.71 Gb of clean data through transcriptomics sequencing to elucidate the response mechanism of maize radicles under TS. Each sample had 6.02 Gb of clean data, with a quality score Q30 of 89.42% or higher and a GC content between 53.07% and 54.32%, indicating that the sequencing data was reliable (Table S1). Principal component analysis (PCA) and correlation heat maps indicated that the three biological replicates clustered closely in the four treatments of the 12 samples, indicating that the transcriptome data had high reproducibility (Figures S1 and S2). Differentially expressed genes (DEGs) were screened based on the fragments per kilobase of exon model per million mapped reads (FPKM) of each gene, with screening criteria of |log_2_FC| ≥ 1 and false discovery rate (FDR) < 0.01. A total of 4894 DEGs were screened in the “ZCT vs. ZTS” comparison group, of which 2379 were upregulated and 2515 were downregulated (Figure 4a; Table S2-1). In the “DCT vs. DTS” comparison group, 5301 DEGs were identified, including 2614 upregulated and 2687 downregulated genes (Table S2-2). A total of 3005 DEGs were co-expressed between the “ZCT vs. ZTS” and “DCT vs. DTS” comparison groups (Figure 4b, Table S2-3).

Gene Ontology (GO) enrichment analysis revealed that these DEGs exhibited changes in enrichment results for molecular function (MF), cellular component (CC), and biological process (BP) (Figure 4c, Figures S3a and S4a). In the top 20 terms of DEGs in the “DCT vs. DTS” comparison group, six GO terms were enriched in BP, whereas DEGs in the “ZCT vs. ZTS” comparison group were enriched in three GO terms. Although maize radicles of different genotypes exhibited varying degrees of responses to TS, they were significantly (*p* < 0.05) enriched terms such as “hydrogen peroxide catabolic process” (GO: 0042744) and “response to oxidative stress” (GO: 0006979) (Table S3).

Kyoto Encyclopedia of Genes and Genomes (KEGG) enrichment analysis indicated that in the common DEGs of “ZCT vs. ZTS” and “DCT vs. DTS,” glutathione metabolism (ko00480), flavonoid biosynthesis (ko00941), isoflavonoid biosynthesis (ko00943), and other pathways were significantly enriched (*p* < 0.05) (Figure 4d, Table S4-1); whereas the plant–pathogen interaction (ko04626), phenylpropanoid biosynthesis (ko00940), flavonoid biosynthesis, and isoflavonoid biosynthesis pathways were significantly enriched (*p* < 0.05) only in the “ZCT vs. ZTS” comparison group (Figure S3b, Table S4-2). Phenylpropanoid biosynthesis, flavonoid biosynthesis, starch and sucrose metabolism (ko00500), isoflavonoid biosynthesis, and other pathways were significantly enriched (*p* < 0.05) only in the “DCT vs. DTS” comparison group (Figure S4b, Table S4-3). These results preliminarily indicate that the phenylpropanoid biosynthesis, flavonoid biosynthesis, and isoflavonoid biosynthesis pathways actively participate in the response of maize radicles to TS.

### 2.4. Co-Expression Trend Analysis of DEGs in Maize Radicles Under TS

**Figure 5:**
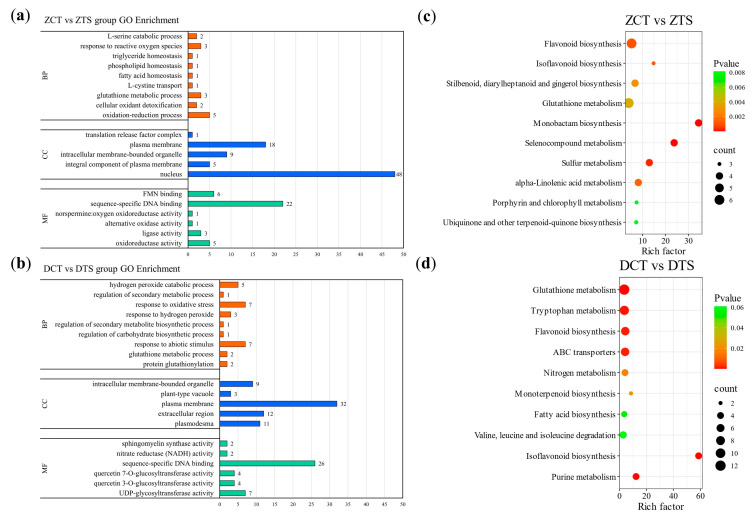
GO and KEGG enrichment analysis of DEGs in co-expression trend modules across different treatment groups. (**a**,**b**) show the GO enrichment analysis of DEGs in the modules of ZCT vs. ZTS” and “DCT vs. DTS”, respectively; (**c**,**d**) represent the KEGG enrichment analysis of DEGs in ZCT vs. ZTS” and “DCT vs. DTS”, respectively. In the bubble chart, the x-axis represents the enrichment factor, and the y-axis represents the KEGG pathway. A larger enrichment factor indicates a higher degree of enrichment; a larger point indicates a greater number of DEGs in the pathway; and a redder point indicates more significant enrichment. ZCT: control treatment of ZD958; ZTS: TS treatment of ZD958; DCT: control treatment of DMY1; DTS: TS treatment of DMY1; MF: molecular function; CC: cellular component.

Based on RNA-Seq data, co-expression trend analysis was performed on the identified DEGs. The 4894 DEGs of ZD958 after TS were divided into 11 modules, and the 5301 DEGs of DMY1 were divided into 15 modules. The DEGs in modules 1, 3, 4, 7, 9, 10 in the “ZCT vs. ZTS” comparison group, and modules 2, 5, 6, 7, 8, 9, 12, and 15 in the “DCT vs. DTS” comparison group showed an upward trend (Figure S5a,b). In these modules, the DEGs in modules 4 and 10 of the “ZCT vs. ZTS” comparison group were associated with antioxidant defense and ROS clearance. GO enrichment analysis showed that significantly enriched (*p* < 0.05) terms in BP included “oxidation-reduction process” (GO:0055114), “cellular oxidant detoxification” (GO:0098869), and “response to reactive oxygen species” (GO:0000302). In MF, significantly enriched (*p* < 0.05) terms included “oxidoreductase activity” (GO:0016491) and “alternative oxidase activity” (GO:0009916) (Figure 5a, Table S5-1).

KEGG enrichment analysis revealed that pathways such as flavonoid biosynthesis, isoflavonoid biosynthesis, and selenocompound metabolism (ko00450) were significantly enriched (*p* < 0.05, Figure 5c, Table S5-2). The DEGs in modules 8 and 9 of the “DCT vs. DTS” comparison group were primarily related to secondary metabolism and antioxidant defense. GO enrichment analysis revealed significant (*p* < 0.05) enrichment in BP for entries such as “response to hydrogen peroxide,” “response to oxidative stress,” “regulation of secondary metabolic process” (GO:0043455), and hydrogen peroxide catabolic process (Figure 5b, Table S5-3); KEGG pathways such as flavonoid biosynthesis, monoterpenoid biosynthesis (ko00902), and isoflavonoid biosynthesis were significantly enriched (*p* < 0.05, Figure 5d, Table S5-4). This result further indicates that pathways such as flavonoid biosynthesis and isoflavonoid biosynthesis are potential candidate pathways for investigating the response of maize radicles to TS.

### 2.5. Weighted Gene Co-Expression Network Analysis of DEGs in Maize Radicles Under TS

**Figure 6:**
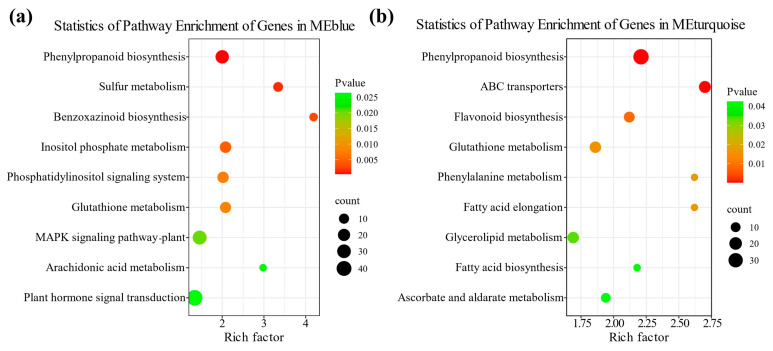
WGCNA and KEGG enrichment analysis of DEGs in different treatment comparison groups. (**a**) KEGG enrichment analysis of DEGs in the MEblue module of WGCNA; (**b**) KEGG enrichment analysis of DEGs in the MEturquoise module of WGCNA. In the bubble plot, the x-axis represents the enrichment factor, and the y-axis represents the KEGG pathway. A higher enrichment factor indicates a greater degree of enrichment; larger points indicate a greater number of DEGs in the pathway; and redder points indicate more significant enrichment.

Using weighted gene co-expression network analysis (WGCNA), the DEGs of two different genotypes of maize radicles under TS were integrated into two color modules, MEblue and MEturquoise (Figure S6). The DEGs in the MEblue module were primarily related to the plant antioxidant system and membrane stability, whereas those in the MEturquoise module were mainly related to the antioxidant system and energy supply. KEGG enrichment analysis of DEGs in the two modules revealed that DEGs in the MEblue module were significantly enriched (*p* < 0.05) in pathways such as isoflavonoid biosynthesis, phenylpropanoid biosynthesis, and sulfur metabolism (ko00920) (Figure 6a,b; Table S6-1). DEGs in the MEturquoise module were significantly enriched (*p* < 0.05) in pathways such as phenylpropanoid biosynthesis, ABC transporters (ko02010), and flavonoid biosynthesis (Table S6-2). These results indicate that the phenylpropanoid biosynthesis, flavonoid biosynthesis, and isoflavonoid biosynthesis pathways are key candidate pathways in the transcriptional regulatory network of maize radicle response to TS.

### 2.6. Quantitative Real-Time Polymerase Chain Reaction Analysis of DEGs in Maize Radicles Under TS

**Figure 7:**
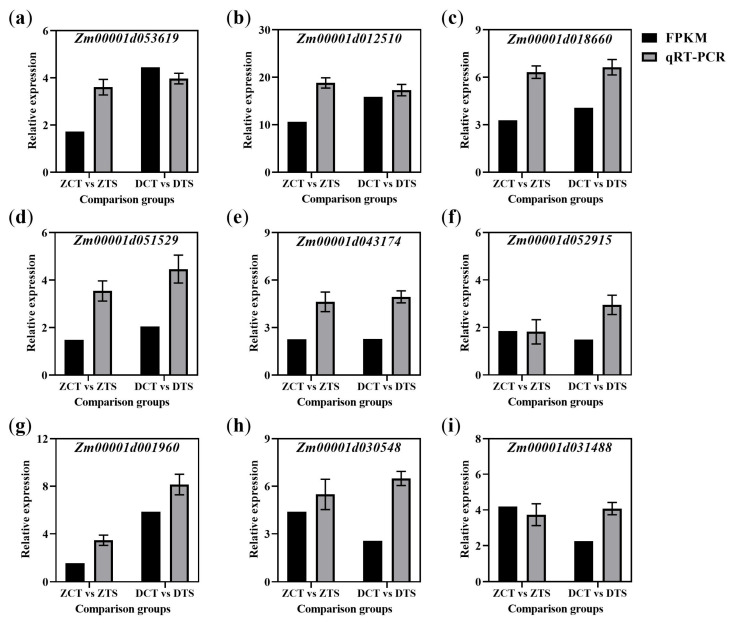
Nine DEGs were randomly selected from the key candidate pathways for qRT-PCR analysis. Relative expression level of (**a**) *Zm00001d053619*; (**b**) *Zm00001d012510*; (**c**) *Zm00001d018660*; (**d**) *Zm00001d051529*; (**e**) *Zm00001d043174*; (**f**) *Zm00001d052915*; (**g**) *Zm00001d001960*; (**h**) *Zm00001d030548*; and (**i**) *Zm00001d031488*. Black represents the FPKM values of DEGs in the transcriptome; gray represents the relative expression levels of DEGs in qRT-PCR. Data represent the mean ± SE from three biological replicates (*n* = 3), with each biological replicate validated by three technical replicates. ZCT: control treatment of ZD958; ZTS: TS treatment of ZD958; DCT: control treatment of DMY1; DTS: TS treatment of DMY1; FPKM: fragments per kilobase of exon model per million mapped reads; qRT-PCR: quantitative real-time polymerase chain reaction.

Key regulatory pathways actively involved in the response of maize seed radicles to TS during the seed germination stage were comprehensively screened using DEGs analysis, co-expression trend analysis, and WGCNA. These pathways included phenylpropanoid biosynthesis, flavonoid biosynthesis, and isoflavonoid biosynthesis. Nine genes were randomly selected from these pathways for quantitative real-time polymerase chain reaction (qRT-PCR) analysis. Although the qRT-PCR values of the selected genes differed slightly from the FPKM values in RNA-Seq, the gene expression trends between the two were consistent, thereby validating the reliability of the RNA-Seq data (Figure 7).

### 2.7. Metabolomics Analysis of Maize Radicles Under TS

Metabolomics analysis was performed on radicle samples under different treatments to analyze their metabolic changes during germination in response to TS. PCA showed that, compared with CT treatment, TS treatment resulted in significant differences in metabolite levels among different genotypes of maize radicles, and all biological replicates exhibited good reproducibility (Figure S7). Further screening and comparison of differentially accumulated metabolites (DAMs) between different treatment groups identified 931 DAMs (574 upregulated and 357 downregulated) in the “ZCT vs. ZTS” comparison group and 1194 DAMs (554 upregulated and 640 downregulated) in the “DCT vs. DTS” comparison group. Additionally, 544 common DAMs were identified that responded to TS in both maize varieties (Figure S8).

KEGG enrichment analysis of DAMs revealed that pathways such as isoquinoline alkaloid (ko00950), isoflavonoid, and flavone and flavonol biosynthesis (ko00944) were enriched in the top 20 in the “ZCT vs. ZTS” comparison group (Table S7-1). Flavonoid biosynthesis, isoflavonoid biosynthesis, flavone and flavonol biosynthesis pathways were enriched in the top 20 in the “DCT vs. DTS” comparison group (Table S7-2); The isoflavonoid biosynthesis, phenylalanine metabolism (ko00360), and phenylpropanoid biosynthesis pathways were enriched in the top 20 common DAMs in both comparison groups (Table S7-3). The results of comprehensive transcriptomic and metabolomic analyses demonstrate that the phenylpropanoid, flavonoid biosynthesis, isoflavonoid biosynthesis, and flavone and flavonol biosynthesis pathways may play crucial roles in regulating maize radicle response to TS.

### 2.8. Analysis of the Regulatory Pathway of Flavonoid Metabolism in Maize Radicles Under TS

**Figure 8:**
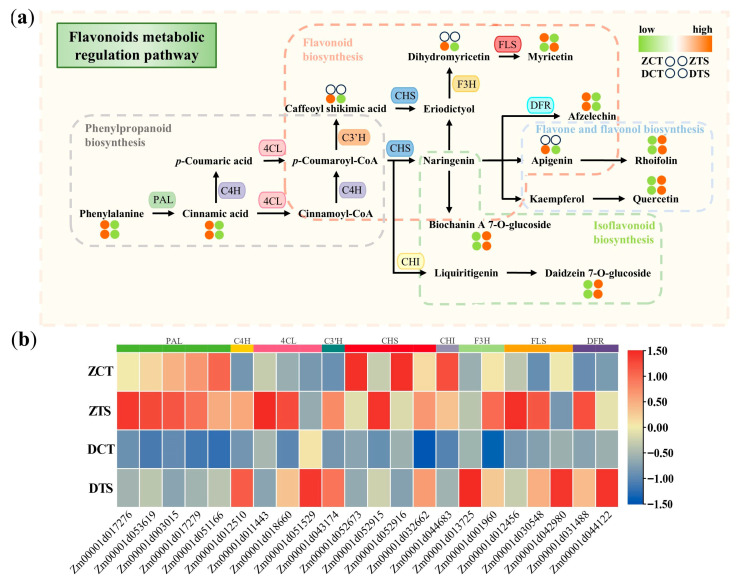
Analysis of the metabolic regulation pathways of flavonoids in maize radicles under different treatment conditions. (**a**) Trends in changes in related metabolites in maize radicles of different genotypes under TS. The gray dashed box indicates phenylpropanoid biosynthesis, the orange dashed box indicates flavonoid biosynthesis, the green dashed box indicates isoflavonoid biosynthesis, and the blue dashed box indicates flavone and flavonol biosynthesis. Red to green indicates DAM content from high to low; (**b**) transcriptional changes in genes related to flavonoid compound metabolism in maize radicles of different genotypes under TS. Red to blue indicates DEG expression from high to low. PAL: phenylalanine ammonia-lyase; C4H: trans-cinnamate 4-monooxygenase; 4CL: 4-coumarate-CoA ligase; C3′H: 5-O-(4-coumaroyl)-D-quinate 3′-monooxygenase; CHS: chalcone synthase; CHI: chalcone isomerase; F3H: naringenin 3-dioxygenase; FLS: flavonol synthase; DFR: bifunctional flavanone 4-reductase; ZCT: control treatment of ZD958; ZTS: TS treatment of ZD958; DCT: control treatment of DMY1; DTS: TS treatment of DMY1.

**Figure 9:**
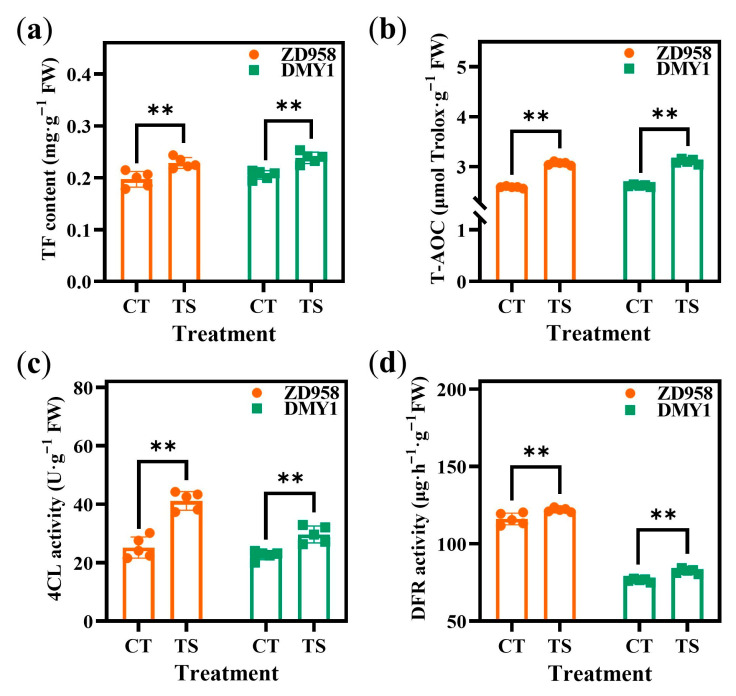
Changes in TF content, total antioxidant capacity of maize radicles and the activity of key enzymes in the flavonoid metabolic pathway under different treatments. (**a**) TF content; (**b**) T-AOC; (**c**) 4CL activity; and (**d**) DFR activity in maize radicles of different genotypes under CT or TS treatment conditions. CT: control treatment; TS: low-temperature stress; TF: total flavonoid; T-AOC: total antioxidant capacity; 4CL: 4-coumarate-CoA ligase; DFR: bifunctional dihydroflavonol 4-reductase. Data represent the mean ± SE of five replicates (*n* = 3). **: *p* < 0.01.

Based on transcriptomic and metabolomic data, this study constructed an integrated pathway of key metabolic regulatory pathways in maize radicles responding to TS, including phenylpropanoid biosynthesis (ko00940), flavonoid biosynthesis (ko00941), isoflavonoid biosynthesis (ko00943), and flavone and flavonol biosynthesis (ko00944). In the phenylpropanoid biosynthesis pathway, genes encoding phenylalanine ammonia-lyase (PAL), trans-cinnamate 4-monooxygenase (C4H), and 4-coumarate-CoA ligase (4CL), such as *Zm00001d053619*, *Zm00001d012510*, and *Zm00001d018660*, were upregulated, which may enhance the activity of the corresponding enzymes and promote phenylalanine (Phe), cinnamic acid, and *p*-coumaric acid synthesis (Figure 8a,b). Similarly, in the flavonoid biosynthesis pathway, genes encoding chalcone synthase (CHS) and bifunctional dihydroflavonol 4-reductase (DFR), such as *Zm00001d052915* and *Zm00001d031488*, were upregulated, leading to an increase in the metabolic rate of afzelechin. The upregulation of *Zm00001d001960* and *Zm00001d030548*, which encode naringenin 3-dioxygenase (F3H) and flavonol synthase (FLS), induced changes in myricetin and dihydromyricetin content. In addition, the content of metabolites such as quercetin, daidzein 7-O-glucoside, biochanin A 7-O-glucoside, apigenin, and rhoifolin in isoflavonoid biosynthesis, and the flavone and flavonol biosynthesis pathways also changed in response to changes in the expression levels of related enzyme genes induced by TS. However, in the integration pathway, the content changes of 5-O-caffeoyl shikimic acid, apigenin, dihydromyricetin, and myricetin differed between ZD958 and DMY1 radicles, which may be owing to differences in the expression intensity or pattern of these compounds in response to TS between the two maize genotypes. Measuring the antioxidant capacity of maize radicles and the activity differences in key enzymes in the flavonoid metabolic regulation pathway under different treatment conditions indicated that, compared to CT treatment, ZTS and DTS increased total flavonoid (TF) content in radicle by 15.99% and 16.01%, respectively, and total antioxidant capacity (T-AOC) by 18.41% and 18.54%, respectively. 4CL ligase activity increased by 63.54% and 31.16%, respectively, and DFR activity increased by 5.09% and 7.68%, respectively. All differences reached the level of extremely significant difference (*p* < 0.01; Figure 9).

## 3. Discussion

This study selected representative corn varieties widely cultivated in the high-latitude cold regions of northeastern China as research subjects. Previous studies [24,28] commonly used 5 °C as the TS treatment condition and used the suitable temperature of 15 °C during germination after spring sowing in the high-latitude cold regions of northeastern China as the CT condition [24,29] to investigate the effects of TS on the growth of maize radicles during germination. In this study, radicle growth in ZD958 and DMY1 was inhibited under TS treatment, specifically manifested by a significant reduction in terminal radicle length, growth increment, total FW, and total DW. Low temperatures [30,31,32], drought [33,34,35], salinity [30,36,37], and waterlogging [38,39,40] are typical adverse environmental conditions in farmland. Zhang et al. [32] conducted low-temperature germination ability assessment tests on 222 maize inbred lines and observed significant differences in the length of maize radicles among different genotypes. They concluded that radicle growth performance can be used as a key indicator for evaluating the low-temperature tolerance of maize. TS treatment led to an increase in the accumulation of ROS, resulting in elevated levels of O_2_^−^, H_2_O_2_, and MDA in maize radicles, as well as an increase in REC. These differences were highly significant compared to CT treatment, consistent with previous studies on flower organs of *Pyrus hopeiensis* [41], intact seeds of waxy maize during germination [42], and young leaves of *Phaseolus vulgaris* L. [43].

SOD, POD, CAT, and APX are key indicators for assessing plant cold sensitivity [24,44,45,46]. SOD serves as the first line of defense against ROS damage in plant cells, catalyzing the dismutation of superoxide anion radicals into H_2_O_2_ [47]. H_2_O_2_ is then catalyzed by CAT [48], POD [49], and APX [50] to form H_2_O and O_2_, thereby reducing ROS-induced oxidative damage and enhancing plant stress resistance. In this study, the activities of relevant antioxidant enzymes in the radicles after TS treatment were significantly higher than those after CT treatment. These results indicate that the TS conditions simulated in this study caused metabolic imbalance and oxidative damage of ROS in the radicle cells, inhibiting normal radicle growth. Furthermore, the defense mechanism of the antioxidant system was activated, which can be further used for integrated analysis of transcriptomics and metabolomics to elucidate the regulatory pathways of maize radicle response to TS at the molecular level during germination.

Numerous studies have applied RNA-Seq technology to explore the DEGs and their regulatory mechanisms in different plant tissues, such as the leaf veins and petioles of *Broussonetia papyrifera* [51], leaves of *Camellia sinensis* [52], and leaves and roots of *Beta vulgaris* [53], in response to TS. Candidate genes can be screened for in RNA-Seq data using various methods, including DEG analysis, co-expression trend analysis, and WGCNA [54,55]. GO and KEGG are often used to explore the functions of DEGs [56]. In this study, after TS treatment, significant enrichment of GO terms such as “response to hydrogen peroxide,” “response to reactive oxygen species,” “hydrogen peroxide catabolic process,” “response to oxidative stress,” “oxidation-reduction process,” “oxidoreductase activity,” and “alternative oxidase activity” was observed in the embryonic roots of different maize genotypes. Additionally, KEGG pathways such as phenylpropanoid biosynthesis, flavonoid biosynthesis, and isoflavonoid biosynthesis were significantly enriched. These pathways are also actively involved in cold stress responses in *Citrus* [57] and *Fagopyrum tataricum* [58].

Metabolomics provides insights into the entire metabolic process of plants and validates the accuracy of transcriptomics [59]. Flavonoids are important compounds for the cold tolerance and adaptation of *Arabidopsis*, and their complete loss or significant reduction can impair cold resistance mechanisms [60]. For example, after cold treatment, DAMs in *Musa* spp. leaves were significantly enriched in the flavone and flavonol biosynthesis and valine, leucine, and isoleucine degradation pathways [61]. Similarly, DAMs in the leaves of *Liriope spicata* were significantly enriched in pathways related to carbon fixation in photosynthetic organisms, flavone and flavonol biosynthesis, and flavonoid biosynthesis [62]. However, in this study, DAMs were primarily enriched in pathways such as isoflavonoid biosynthesis, flavone and flavonol biosynthesis, and phenylpropanoid biosynthesis, and the levels of DAMs such as quercetin, rhoifolin, and afzelechin also changed significantly. These results indicate that after perceiving changes in ROS levels, the radicle activates the antioxidant defense system, which is manifested in two ways: on the one hand, it enhances the activity of antioxidant enzymes to rapidly clear ROS and alleviate acute oxidative damage; on the other hand, it initiates metabolic enhancement mechanisms, providing raw materials through the phenylpropanoid biosynthesis pathway to continuously synthesize potent antioxidant substances such as flavonoids. These flavonoids enhance the precision of ROS clearance, strengthen oxidative stress-buffering capacity, and synergistically regulate ROS homeostasis with antioxidant enzymes [63]. This process demonstrates that the flavonoid metabolic regulation pathway, including phenylpropanoid biosynthesis, flavonoid biosynthesis, isoflavonoid biosynthesis, and flavone and flavonol biosynthesis, may play a pivotal role in the response of maize radicles to TS.

Based on the integrated pathway constructed according to the key metabolic regulatory pathways of maize radicles in response to TS, this study found that the expression patterns of key enzyme genes involved in flavonoid metabolism differed among different genotypes of maize radicles, which may lead to differences in flavonoid metabolic activity. Under ZCT, flavonoid synthesis was more active, potentially indicating stronger “metabolic reserves.” In contrast, DCT is relatively conservative, reserving more regulatory space in response to low temperatures. As a key pathway in the synthesis of flavonoids, PAL and C4H catalyze the conversion of Phe into cinnamic acid and *p*-coumaric acid [64], which are then converted into *p*-coumaroyl-CoA by 4CL and C4H. Under ZTS and DTS conditions, the genes related to PAL, C4H, and 4CL were upregulated, promoting the flow of precursors in the phenylpropanoid biosynthesis pathway and providing sufficient raw materials for flavonoid synthesis. Under ZTS, CHS catalyzes *p*-coumaroyl-CoA to form naringenin [65], and F3H and FLS are significantly upregulated, directing the conversion of naringenin into flavonols, such as quercetin [66,67]. However, although the CHS gene is also upregulated in DTS, the extent of upregulation is weaker than that in ZTS, and the expression of enzymes related to isoflavone synthesis, such as CHI, is more balanced. This finding suggests that DMY1 radicles do not rely on the high-intensity synthesis of a single flavonol but instead expand antioxidant capacity through multiple isoflavones, helping radicles balance metabolism and adapt to low temperatures, reflecting a different response to low temperatures compared to ZD958 and compensating for the insufficient intensity of flavonol synthesis in DMY1.

**Figure 10:**
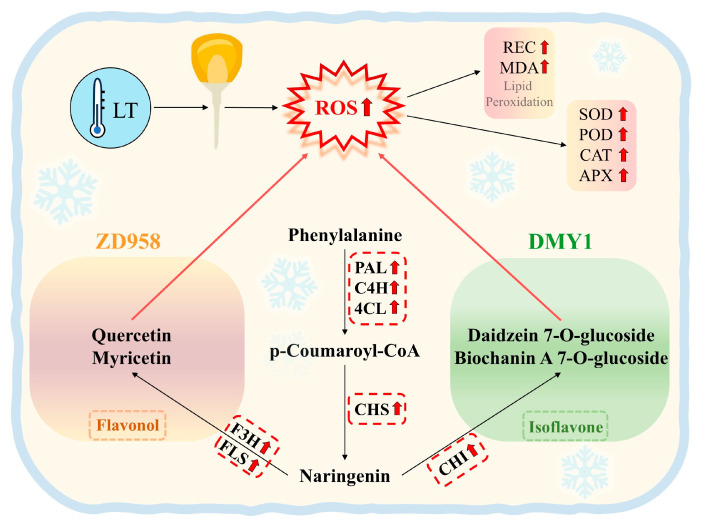
Mechanism diagram of flavonoid metabolism regulation in maize radicles under TS. LT: low temperature; ROS: reactive oxygen species, including O_2_^−^ and H_2_O_2_; REC: relative electrolytic conductivity; MDA: malondialdehyde; SOD: superoxide dismutase; POD: peroxisome; CAT: catalase; APX: ascorbate peroxidase; PAL: phenylalanine ammonia-lyase; C4H: trans-cinnamate 4-monooxygenase; 4CL: 4-coumarate-CoA ligase; CHS: chalcone synthase; F3H: naringenin 3-dioxygenase; FLS: flavonol synthase; CHI: chalcone isomerase. Black thin arrows indicate the direction of action or metabolic flux, red short arrows indicate increased content/activity or upregulated related genes, red long arrows emphasize the direction of action, and ZD958 and DMY1 are represented by boxes of different colors to show their varietal differences.

In summary, the maize radicles of ZD958 and DMY1 respond to TS by sensing changes in ROS content, activating the supply of raw materials and core regulation of the flavonoid metabolic pathway, and allocating intermediate metabolites to antioxidant functions. Different genotypes exhibit distinct but equally effective low-temperature response mechanisms, such as “flavonol-dominated (ZD958)” and “isoflavone-coordinated (DMY1).” However, their essence lies in adaptive strategies of the antioxidant system, ultimately enabling ROS scavenging, redox balance maintenance, and the construction of an antioxidant barrier to ensure basal metabolism and growth development of the radicles under low-temperature conditions. This may be one of the key pathways for maize radicles to respond to TS (Figure 10). This study revealed statistically significant correlations between gene expression levels in integrated pathways (e.g., *Zm00001d051529*) and multiple phenotypic/physiological indicators through combined transcriptome and metabolome analysis of two different genotypes (Figure S9). However, this alone does not directly prove that flavonoid metabolic regulation pathways play a key role in maize radicle responses to TS. Subsequent validation will include targeted enzyme inhibition experiments (screening for the pathway enzyme most strongly correlated with phenotypes and matching specific inhibitors to assess the functional necessity of core pathway enzymes); mutation-based validation experiments (constructing overexpression and knockout lines and comparing pathway and phenotypic differences with wild-type plants to validate genetic necessity and sufficiency of core pathway genes); and multi-varietal field validation trials to further elucidate the regulatory mechanisms of the flavonoid metabolic pathway.

## 4. Materials and Methods

### 4.1. Test Varieties and Experimental Design

The maize varieties ZD958 (Zhengdan958, dent maize, developed by the Institute of Grain Crops, Henan Academy of Agricultural Sciences, Zhengzhou, China) and DMY1 (Demeiya1, flint maize, developed by Beidahuang Kenfeng Seed Industry Co., Ltd., Harbin, China), which are primarily cultivated in the high-latitude cold regions of northeastern China, were selected. Seeds were harvested in 2023, dried according to local standard DB23/T 1036-2006, stored under vacuum at 4 °C, and maintained at a germination rate ≥95%. Uniform and consistent seeds across all varieties were selected. Prior to experimenting, all seeds were disinfected in a 1% NaClO solution for 20 min, surface-disinfected in a 75% (*v*/*v*) ethanol solution for 60 s, and rinsed with sterile water six times. The disinfected seeds were placed in paper bed germination boxes (14 cm × 14 cm × 6 cm) containing 10 mL of sterile water. Each germination box contained 25 seeds, which were placed in an artificial climate chamber (DRX-330E, Ningbo Dongnan Instrument Co., Ltd., Ningbo, China) at a temperature of 25 ± 0.5 °C and relative humidity of 50 ± 5% for dark cultivation. The climate chamber used was calibrated within 1 week prior to the start of the experiment. After 60 h of germination, seeds with consistent radicle lengths were selected and transplanted into new germination trays (10 seeds per tray). Dark cultivation was continued for 48 h under conditions of 15 ± 0.5 °C (CT) and 5 ± 0.5 °C (TS). For ZD958, the treatments are denoted as ZCT and ZTS, respectively. Similarly, the treatments for DMY are denoted as DCT and DTS, respectively.

### 4.2. Radicle Length, Growth Increment, FW, and DW Measurement

Before CT or TS treatment, the initial radicle length of all germinating seeds in each germination box was measured, and the average value was recorded as *RL*_1_; after 48 h of cultivation under CT or TS treatment conditions, the terminal radicle length of all germinating seeds in each germination box was measured. The average value was recorded as *RL*_2_, and radicle growth increment was determined as *RG* = *RL*_2_ − *RL*_1_.

Maize radicles from the two maize varieties were collected after treatment with CT or TS. The total FW of 10 maize radicles in each germination box was measured using a Presica LS120A balance (Sartorius AG, Goettingen, Germany). The radicles were then fixed at 105 °C for 30 min and dried at 80 °C until constant weight, after which the total DW of all radicles in each germination tray was measured.

### 4.3. Physiological and Biochemical Indicator Measurement

#### 4.3.1. Determination of REC and MDA Content in Maize Radicles

Surgical scissors were used to cut maize radicles along the radicle sheath from the germination boxes of each treatment group, as previously described [68] with slight modifications. A 0.1 g sample was taken as one replicate, with five replicates per treatment. The radicle samples were repeatedly rinsed with distilled water and then transferred to centrifuge tubes containing 5 mL of distilled water. After 24 h, the initial conductivity (denoted as *R*_1_) was measured at 25 °C. The centrifuge tubes containing the samples were then placed in a boiling water bath for 30 min, cooled to room temperature, and the final conductivity (denoted as *R*_2_) was measured as *REC* = *R*_1_/*R*_2_ × 100%. MDA content was determined according to the method of Hodges et al. [69].

#### 4.3.2. Qualitative and Quantitative Analysis of O_2_^−^ and H_2_O_2_

The distribution of O_2_^−^ was detected using NBT staining [70]. The O_2_^−^ content was determined using the hydroxylamine oxidation method [71]. 3,3′-diaminobenzidine histochemical staining was performed as previously described [72] with modifications to observe H_2_O_2_ accumulation. Quantitative measurement of H_2_O_2_ was performed using the colorimetric method as previously described [73], with slight modifications. Maize radicles (0.5 g) were homogenized in pre-chilled ethanol and immediately centrifuged at 12,000× *g* for 15 min at 4 °C. Concentrated hydrochloric acid and 25% ammonium hydroxide (0.2 mL) were added to 1 mL of the supernatant to precipitate the peroxide-titanium complex; the precipitate was dissolved in 2 mmol/L H_2_SO_4_, and the absorbance was measured at 415 nm.

#### 4.3.3. Antioxidant Enzyme Activity Measurement

The antioxidant enzyme activity assay was performed as previously described [74]. SOD [EC 1.15.1.1], POD [EC 1.11.1.7], CAT [EC 1.11.1.6], and APX [EC1.11.1.1] activities were determined separately using the Specord Plus 210 spectrophotometer (Analytik Jena AG, Jena, Germany). The SOD activity was measured using the nitroblue tetrazolium method [75], the POD activity was measured using the guaiacol method [76], the CAT activity was measured as previously described [77], and the APX activity was measured as previously described [78].

#### 4.3.4. Determination of TF Content, T-AOC, and 4CL and DFR Activity

TF content, T-AOC, and 4CL and DFR activities in maize radicles were determined using the TF, T-AOC, 4CL, and DFR assay kits (G0118F, G0142F, G1003F24, G1008F, Grace Biotechnology Co., Ltd., Suzhou, China), respectively.

### 4.4. RNA Extraction, Library Construction, and RNA-Seq

Three biological replicates of radicle samples were collected from different maize varieties under CT and TS treatments, for a total of 12 samples. Total plant RNA was extracted using the RNAprep Pure Plant Kit (Tiangen Biotech Co., Ltd., Beijing, China) according to the manufacturer’s instructions. cDNA library construction and sequencing were performed at Biomarker Technologies (Beijing, China) for all test samples. All filtered clean reads were aligned to the maize reference genome (B73_RefGen_v4) using HISAT2 [79]. The alignment results were assembled using String Tie [80]. FPKM [81] was used to quantify gene expression levels to validate the transcriptional expression levels of all samples. The FPKM for each gene was calculated based on gene length and the number of reads mapped.

DESeq2 [82] was used to perform differential analysis between groups to identify DEGs between different treatments. Fold change (FC) ≥ 2 and FDR < 0.05 were used as screening criteria. FC refers to the ratio of expression levels between two sample groups. FDR was obtained by correcting the significance *p*-value and indicated the significance of the difference. DEGs were analyzed using the Cluster Profile R software package (version 3.21) through the GO and KEGG databases [83], and GO and KEGG enrichment pathways were screened using a *p* < 0.05 standard. WGCNA was performed using a similarity threshold of 0.25 and a minimum gene number of 30 for modules.

### 4.5. qRT-PCR Analysis

Nine differentially expressed genes were randomly selected for qRT-PCR validation to validate the reliability and reproducibility of the DEGs obtained by RNA-Seq. Total RNA was extracted from each treated root sample using TRIzol^®^ reagent (Invitrogen, Carlsbad, CA, USA). RNA quality was assessed using a NanoDrop spectrophotometer (Thermo Fisher Scientific, Waltham, MA, USA) and 1% agarose gel electrophoresis. Based on the sequences of these nine genes, specific primers for the nine genes were designed using the Primer Design website (https://www.ncbi.nlm.nih.gov) (Supplementary Table S8), with *ZmActin* [84] as the internal control gene. SYBR qPCR Master Mix (TOYOBO Co., Osaka, Japan) was used. qRT-PCR was performed using the Bio-Rad real-time fluorescent quantitative PCR system (Bio-Rad Laboratories Inc., Hercules, CA, USA) [85]. Each treatment included three biological replicate samples (each biological replicate contained three technical replicates), and gene expression levels were calculated using the 2^−∆∆Ct^ method [81].

### 4.6. Metabolomics Analysis

Three biological replicates of radicle samples were collected from different maize varieties under CT and TS treatments, yielding a total of 12 samples. These samples were sent to Biomarker Technologies (Beijing, China) for metabolomics analysis. The LC/MS system (Waters Corporation Milford, MA, USA) used for analysis consists of a Waters Acquity I-Class PLUS ultra-high-performance liquid chromatography system (Waters Corporation Milford, MA, USA) coupled with a Waters Xevo G2-XS QT high-resolution mass spectrometer (Waters Corporation Milford, MA, USA). The column used was purchased from Waters Acquity UPLC HSS T3 column [particle size, 1.8 μm; 2.1 mm (i.d.) × 100 mm (length)]. DAMs were screened using the following criteria: variable importance projection (VIP) > 1, |log_2_FC| ≥ 0.58, and *p* < 0.05.

### 4.7. Statistical Analyses

Each phenotypic and physiological indicator was analyzed five times. The average measurement of all germinated seeds (10 seeds) in a germination box was considered one repetition when measuring the terminal radicle length, growth, FW, and DW. During physiological measurements, sampling was conducted using a simple randomization method (randomly selecting radicles from each treatment germination tray), and the assessment of indicators was performed as an open-label trial. Data analysis was performed using SPSS 22.0 software (IBM, Armonk, NY, USA) for one-way analysis of variance. If significant differences were observed between groups (*p* ≤ 0.05), Duncan’s test was further applied for multiple comparisons. Annotations for extremely significant differences (*p* ≤ 0.01) are based on the same multiple comparison results. Bivariate Pearson correlation analysis was employed to reveal correlations between genes and physiological indicators, with bilateral t-tests used to assess the significance of these correlations. Data visualization was performed using GraphPad Prism 8.0 (GraphPad Software, Boston, MA, USA), Tbtools v2.012 (https://github.com/CJ-Chen/TBtools/releases, accessed on 20 January 2025), and the Bioinformatics Online Analysis Platform (https://www.bioinformatics.com.cn). Quality control, alignment, and DEG screening of the transcriptomic raw data were performed on the BMKCloud Platform (Biomarker Technologies, Beijing, China). All sequencing data were uploaded to the National Center for Biotechnology Information (login number: PRJNA1050059, Supplementary Table S9).

## 5. Conclusions

TS conditions cause metabolic imbalance and oxidative damage of ROS in radicle cells, inhibiting normal radicle growth, and activating the defense mechanisms of the antioxidant system. The metabolic regulation pathway of flavonoids (phenylpropanoid biosynthesis, flavonoid biosynthesis, isoflavonoid biosynthesis, and flavone and flavonol biosynthesis) may play a pivotal role in maintaining ROS homeostasis in maize radicles. Although maize radicles of different genotypes adopt differentiated flavonoid metabolic regulation strategies—either “flavonol-dominated” or “isoflavone-coordinated”—in response to TS, they follow the common regulatory logic of “low-temperature signal-driven—antioxidant redirection—functional synergy.” The findings of this study contribute to a deeper understanding of the molecular mechanisms underlying the radicle resistance to TS during maize seed germination and provide a theoretical foundation for enhancing the low-temperature adaptability of maize during germination in cold regions. However, the metabolic regulation mechanisms of flavonoids that account for inter-varietal differences under TS require further investigation.

## Data Availability

The data presented in this study are available on request from the corresponding authors. The data are not publicly available due to intellectual property rights.

## Supplementary Materials

The following supporting information can be downloaded at https://www.mdpi.com/article/10.3390/plants14192988/s1, Figure S1: PCA between transcriptomic samples. Figure S2: Correlation analysis between transcriptomic samples. Figure S3: DEGs in “ZCT vs. ZTS” treatment comparison groups and their GO and KEGG enrichment analysis. Figure S4: DEGs in “DCT vs. DTS” treatment comparison groups and their GO and KEGG enrichment analysis. Figure S5: Co-expression trend module classification of DEGs in different treatment comparison groups. Figure S6: Correlation between different WGCNA modules. Figure S7: PCA between metabolome samples. Figure S8: Venn diagram of DAMs in two control groups. Figure S9: Heatmap showing correlations between selected genes in the flavonoid metabolic regulation pathway and physiological/phenotypic traits. Table S1: Sequencing quality control. Table S2: Co-expression of DEGs under control and low temperature stress. Table S3: GO enrichment analysis of two comparison groups. Table S4: KEGG enrichment analysis of two comparison groups. Table S5: GO and KEGG enrichment analysis of DEGs in the co-expression module. Table S6: KEGG enrichment analysis of DEGs in different WGCNA modules. Table S7: KEGG enrichment analysis of DAMs in different comparison groups. Table S8: Primer sequences for qPCR validation. Table S9: Raw data of transcriptome uploaded to NCBI database.

## Abbreviations

The following abbreviations are used in this manuscript:

4CL	4-coumarate-CoA ligase
BP	biological process
C4H	trans-cinnamate 4-monooxygenase
CAT	catalase
CC	cellular component
CHS	chalcone synthase
CT	control
DAB	3,3′-diaminobenzidine
DAM	differentially accumulated metabolites
DEG	differentially expressed gene
DFR	bifunctional dihydroflavonol 4-reductase
DMY1	Demeiya1
DW	dry weight
F3H	naringenin 3-dioxygenase
FC	fold change
FDR	false discovery rate
FLS	flavonol synthase
FPKM	fragments per kilobase of exon model per million mapped reads
FW	fresh weight
GO	Gene Ontology
MDA	malondialdehyde
MF	molecular function
NBT	nitrotetrazolium blue chloride
PAL	phenylalanine ammonia-lyase
PCA	principal component analysis
Phe	phenylalanine
POD	peroxidase
KEGG	Kyoto Encyclopedia of Genes and Genomes
qRT-PCR	quantitative real-time polymerase chain reaction
T-AOC	total antioxidant capacity
TF	total flavonoid
TS	low-temperature stress
SOD	superoxide dismutase
REC	relative electrical conductivity
ROS	reactive oxygen species
WGCNA	weighted gene co-expression network analysis
ZD958	Zhengdan958
