# Supplementary material for: Integrated Transcriptome–Metabolome Analysis Reveals the Flavonoids Metabolism Mechanism of Maize Radicle in Response to Low Temperature
Source: Plants (Basel). 2025 Sep 26;14(19):2988. doi: 10.3390/plants14192988 (PMC12525699; doi:10.3390/plants14192988)
Supplement: Supplementary file 1 [file plants-14-02988-s001.zip › plants-3861315/Supplementary_picture.pdf]

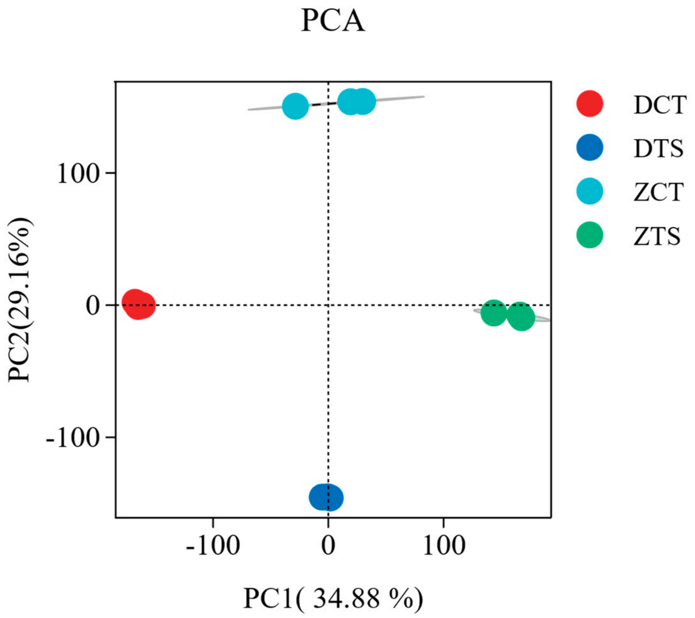

Figure S1 Principal component analysis (PCA) showed the similarity of transcriptome samplesacross treatment conditions.

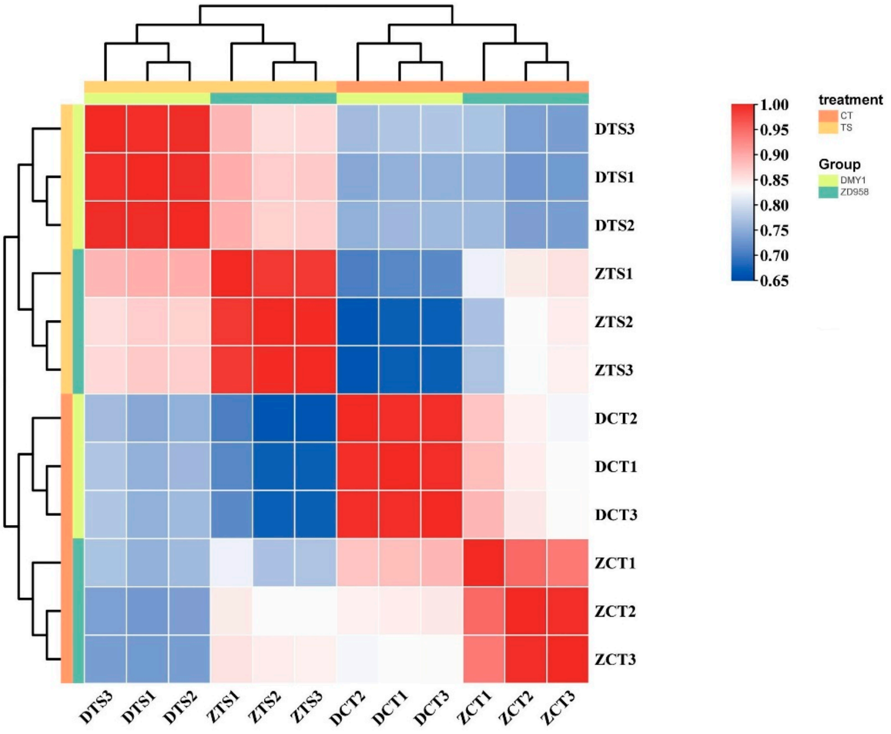

Figure S2 Hierarchical clustering based on transcript expression in each corn embryo root sample and expression level correlations between each pair of samples.

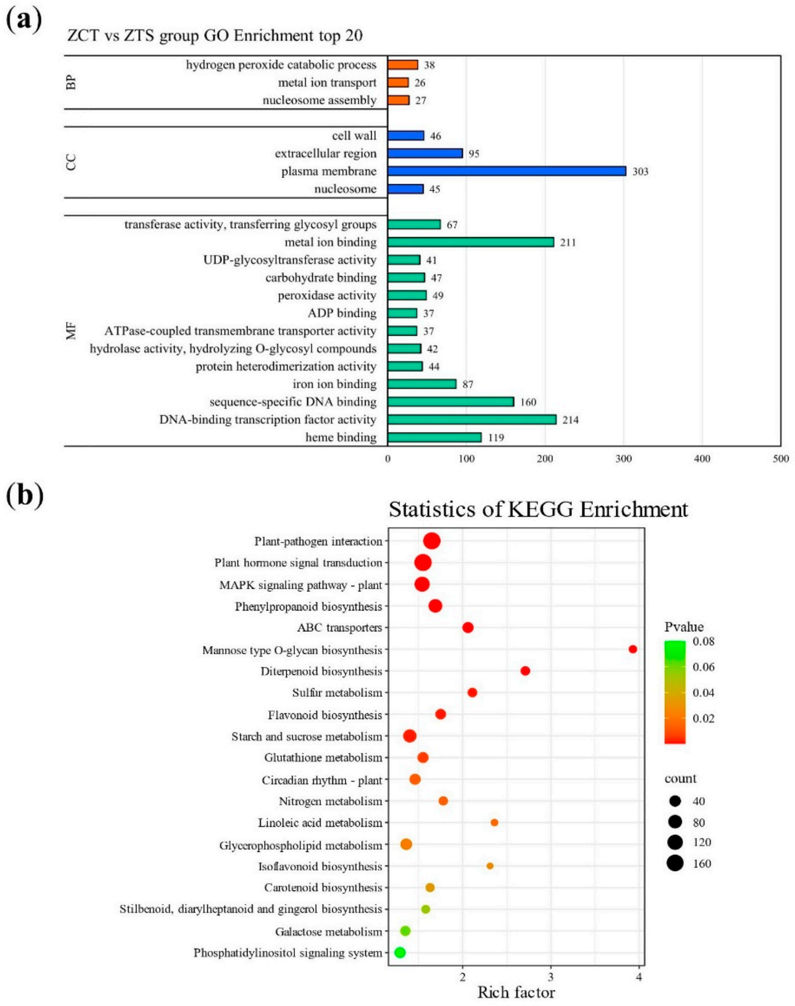

Figure S3 DEGs in "ZCT vs ZTS" treatment comparison groups and their GO and KEGG enrichment analysis. (**a**) Top 20 GO terms analysis of "ZCT vs ZTS"; (**b**) Top 20 KEGG pathway analysis of "ZCT vs ZTS". In the bubble plot, the x-axis represents the enrichment factor, and the y-axis represents the KEGG pathway, with colors ranging from red to green indicating P-values from small to large. ZCT: control treatment of ZD958; ZTS: TS treatment of ZD958; DEGs: differentially expressed genes; GO, Gene Ontology; KEGG: Kyoto En-cyclopedia of Genes and Genomes.

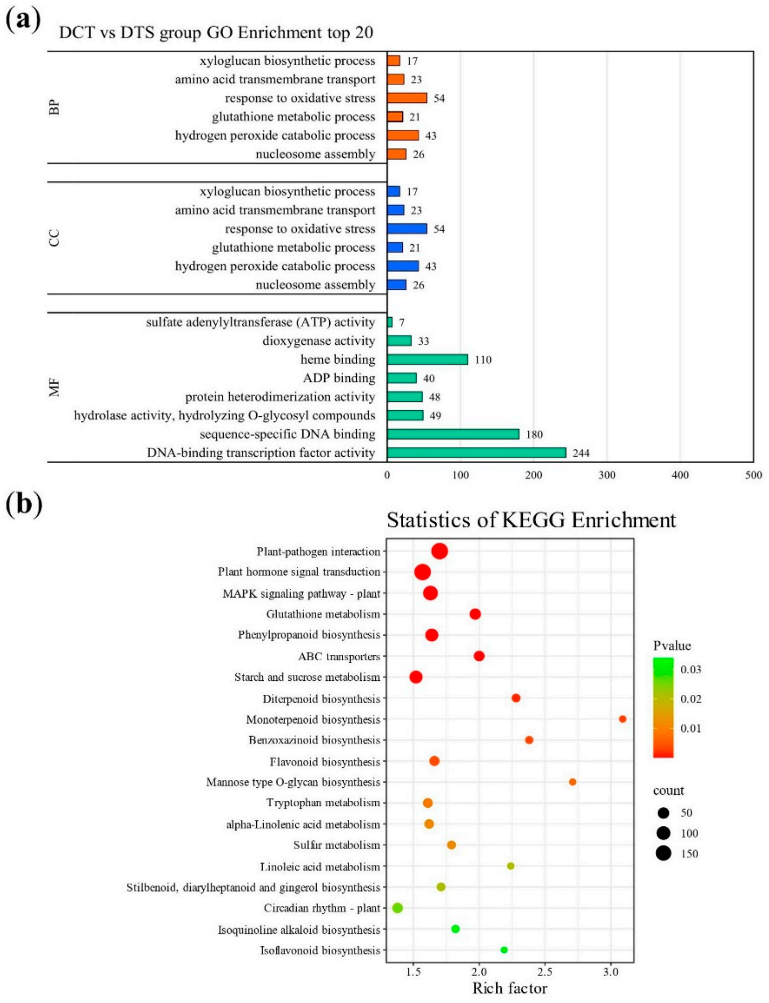

Figure S4 DEGs in "DCT vs DTS" treatment comparison groups and their GO and KEGG enrichment analysis. (**a**) Top 20 GO terms analysis of "DCT vs DTS"; (g, h) Top 20 KEGG pathway analysis of "ZCT vs ZTS" or "DCT vs DTS". In the bubble plot, the x-axis represents the enrichment factor, and the y-axis represents the KEGG pathway, with colors ranging from red to green indicating P-values from small to large. DCT: control treatment of DMY1; DTS: TS treatment of DMY1; DEGs: differentially expressed genes; GO, Gene Ontology; KEGG: Kyoto En-cyclopedia of Genes and Genomes.

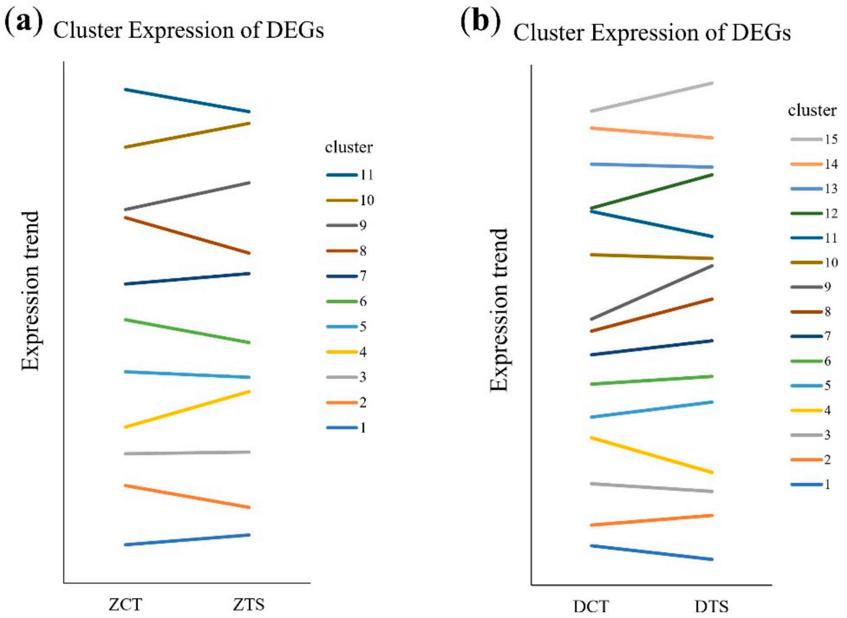

Figure S5 Co-expression trend module classification of DEGs in different treatment comparison groups. (**a**) show the co-expression trends of DEGs in the comparison groups "ZCT vs ZTS"; (**b**) show the co-expression trends of DEGs in the comparison groups "DCT vs DTS".

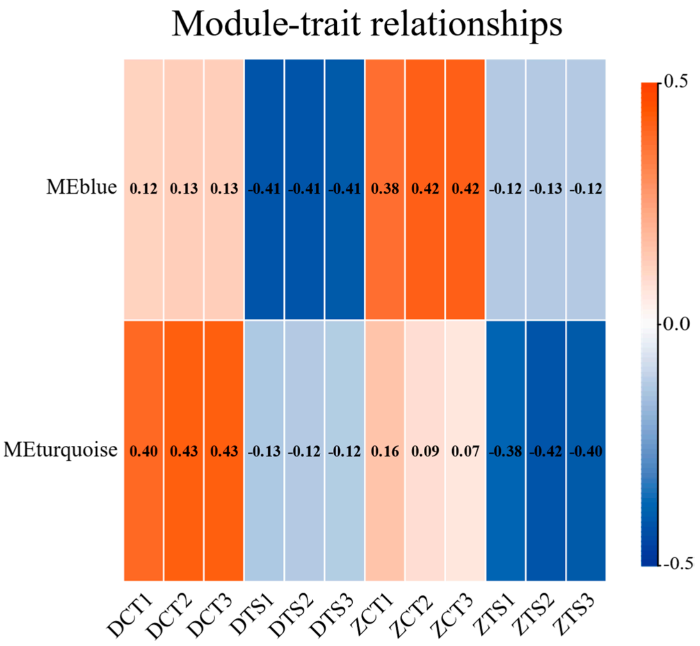

Figure S6 Correlation between transcriptome modules and various treatments in weighted gene co-expression network analysis (WGCNA). The bar chart shows correlation from low to high, from blue to red.

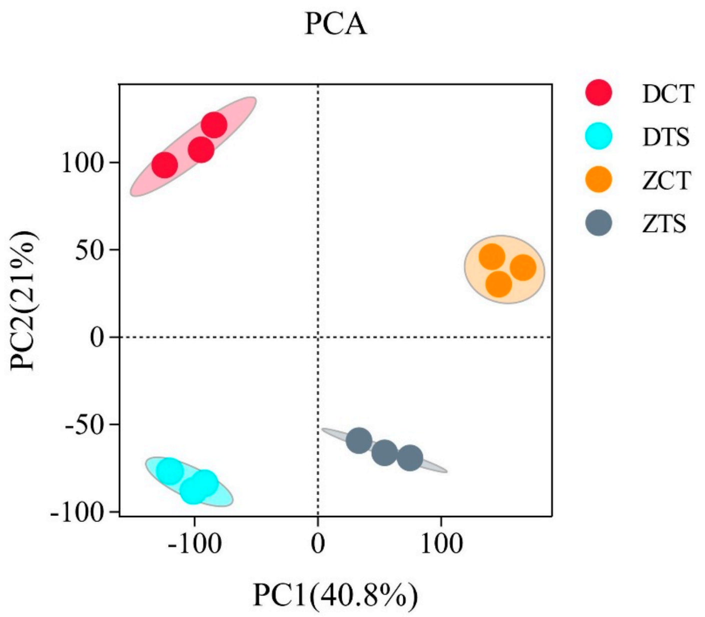

Figure S7 Principal Component Analysis (PCA) showed the similarity of metabolome samples across treatment conditions.

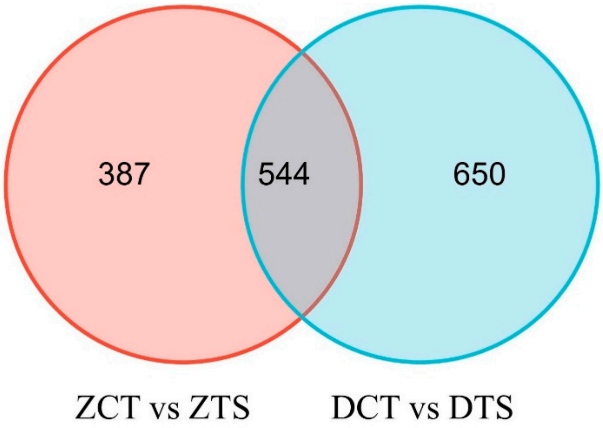

Figure S8 Venn diagrams depicting differential metabolites in response to low temperature in two comparison groups, ZCT vs ZTS and DCT vs DTS embryonic roots.

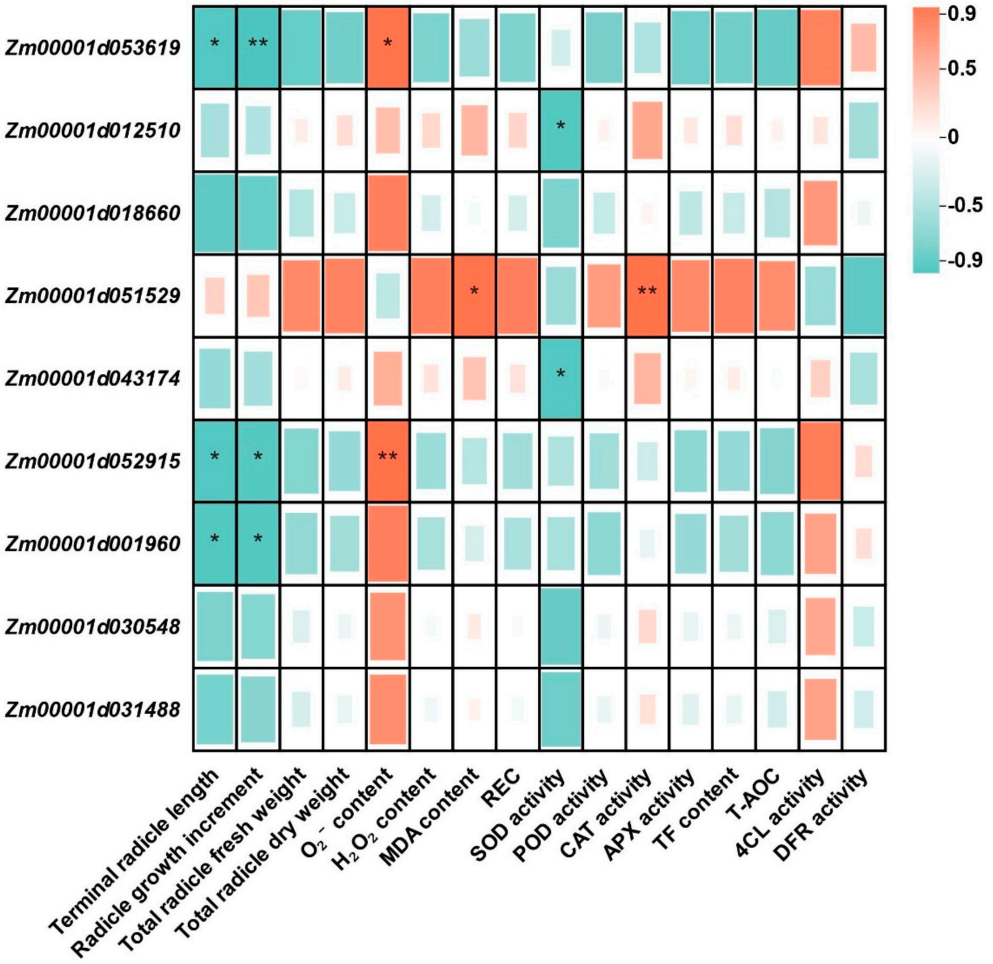

Figure S9 Heatmap showing correlations between selected genes in the flavonoid metabolic regulation pathway and physiological/phenotypic traits. This heatmap displays the Pearson correlation coefficients between each gene and physiological/phenotypic characteristics. Blue tones indicate negative correlations, while red tones indicate positive correlations. *: $P < 0.05$; **: $P < 0.01$.
